# Remote Vital Sensing in Clinical Veterinary Medicine: A Comprehensive Review of Recent Advances, Accomplishments, Challenges, and Future Perspectives

**DOI:** 10.3390/ani15071033

**Published:** 2025-04-03

**Authors:** Xinyue Zhao, Ryou Tanaka, Ahmed S. Mandour, Kazumi Shimada, Lina Hamabe

**Affiliations:** 1 Department of Veterinary Science, Tokyo University of Agriculture and Technology, Tokyo 183-8509, Japan; s239581q@st.go.tuat.ac.jp (X.Z.); ahmedmandour287@gmail.com (A.S.M.); linahamabe@go.tuat.ac.jp (L.H.); 2 Department of Animal Medicine (Internal Medicine), Faculty of Veterinary Medicine, Suez Canal University, Ismailia 41522, Egypt

**Keywords:** remote vital sensing, veterinary medicine, infrared thermography, photoplethysmography, radar sensing, wearable sensors, computer vision, artificial intelligence, animal welfare, non-invasive monitoring

## Abstract

Remote vital sensing in veterinary medicine is a relatively new area of practice, which involves the acquisition of data without invasion of the body cavities of live animals. This paper aims to review several technologies in remote vital sensing: infrared thermography, remote photoplethysmography (rPPG), radar, wearable sensors, and computer vision and machine learning. In each of these technologies, we outline its concepts, uses, strengths, and limitations in multiple animal species, and its potential to reshape health surveillance, welfare evaluation, and clinical medicine in animals. The review also provides information about the problems associated with applying these technologies, including species differences, external conditions, and the question of the reliability and classification of these technologies. Additional topics discussed in this review include future developments such as the use of artificial intelligence, combining different sensing methods, and creating monitoring solutions tailored to specific animal species. This contribution gives a clear understanding of the status and future possibilities of remote vital sensing in veterinary applications and stresses the importance of that technology for the development of the veterinary field in terms of animal health and science.

## Simple Summary

Modern veterinary medicine is exploring new ways to monitor animals’ health without touching them using technologies like thermal cameras, special video systems, radar, wearable devices, and computer analysis. These tools can measure vital signs like heart rate, breathing, and temperature from a distance, which reduces stress for animal patients and allows for continuous health monitoring. While these technologies show great promise for transforming veterinary care, they still need refinement to work effectively across different animal species and environments. Once perfected, these remote monitoring methods could help veterinarians detect and track health issues earlier while keeping their patients more comfortable.

## 1. Introduction

Remote vital sensing in veterinary medicine involves collecting physical and behavioral data using technologies that reduce the need for direct human contact during monitoring. It includes a range of methods from completely hands-off approaches (infrared imaging, radar, and computer vision) to minimally invasive devices (wearable sensors) that send data wirelessly [1,2].

The need for remote vital sensing in veterinary science cannot be overemphasized. The benefit is that it promotes the timely identification of health problems, extends the possibility of chronic disease tracking, and contributes information for research uses [3,4]. Similarly, it is suggested that such technologies may also promote improvements in slippery caressing and reduction in handling and restraint during assessment [5,6].

The field of remote vital sensing in veterinary medicine has grown significantly in recent years, moving from mainly research settings to beginning clinical use [7]. Current research shows several clear patterns that reflect both new technological capabilities and specific veterinary challenges. However, important research gaps still exist. The combination of different technologies into complete monitoring systems remains limited, with most studies examining only one type of sensing method [8]. The research is not equally distributed across animal types, with pets and major farm animals receiving more attention than exotic and wild species [9,10]. There are few clinical studies with enough data to properly validate these methods, and the cost-effectiveness of implementing these technologies in different veterinary settings is not fully evaluated. The developing One Digital Health framework [11] offers an approach for connecting these technologies with broader health systems, though practical applications are still in the early stages.

The current research trends point toward systems that combine multiple types of sensors enhanced by artificial intelligence, merging both contactless and wearable technologies [12]. These developments increasingly focus on clinical testing and practical use across various veterinary environments [13].

This review aims to comprehensively examine the current state of remote vital sensing technologies in veterinary medicine. Throughout the following sections, we will analyze key technologies that are being actively developed and implemented in this field: infrared thermography, remote photoplethysmography (rPPG), radar-based sensing, wearable sensors, and computer vision with machine learning. Each technology will be evaluated for its principles, clinical applications, limitations, and future potential in veterinary practice.

For each of the technologies, we shall outline this information under principles, literature review, and potential applications in veterinary practice. Furthermore, we will discuss the issues arising when implementing these technologies and evaluate possible advancements in their research.

By synthesizing the latest research and identifying key trends, this review seeks to provide veterinary professionals, researchers, and technology developers with a clear understanding of the current landscape and future potential of remote vital sensing in animal healthcare.

## 2. Infrared Thermography

### 2.1. Principles and Technology

**Figure 1:**
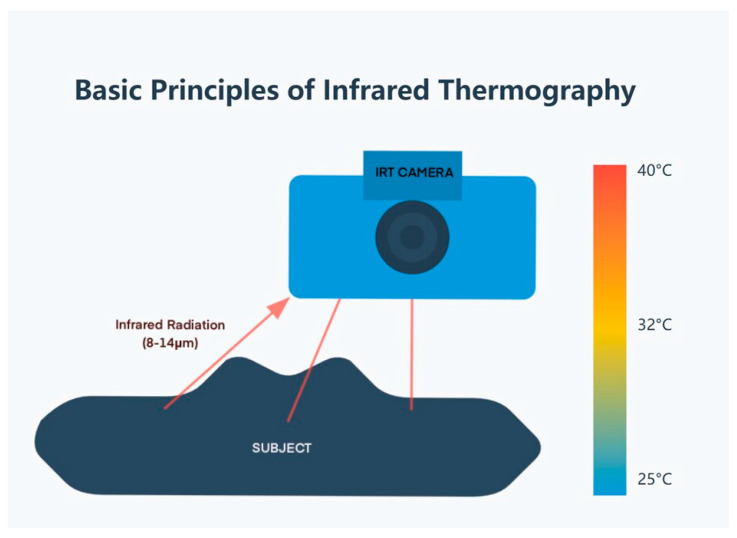
Basic principles of infrared thermography.

Infrared thermography (IRT) is established on the standpoint that all objects with a temperature over absolute zero emit infrared radiation. The radiation emitted is in relation to the temperature of the object and is, hence, capable of delivering non-contact temperature readings [5] (Figure 1). In veterinary uses, IRT cameras monitor and record this infrared radiation emanating from an animal’s skin surface as electrical signals that are worked upon to form a thermograph or thermogram [14].

The current intracutaneous reflectance thermometry cameras employed in veterinary medicine work within the long-wave infrared region (8–14 μm) which is more resistant to absorption by gases in the environment. These cameras can distinguish even the temperatures as small as 0.05 °C making susceptible measurements [15].

The core components of an IRT system include the following:

(1) Infrared detector: Usually a focal plane array (FPA) of microbolometers or quantum detectors.
(2) Optical system: Lenses that are cognizant of the infrared radiation onto the detector.
(3) Signal processing unit: Converts detector signals into temperature values.
(4) Display: Presents the thermal image, frequently using a color palette to symbolize temperature variations.

Recent advancements in IRT have led to the development of portable, high-resolution cameras with real-time imaging capabilities. Some systems now incorporate smartphone compatibility, making them more accessible for field use in veterinary practice [16].

It is important to note that IRT measures surface temperature, which can be influenced by various factors, including ambient temperature, airflow, fur or feather cover, and the animal’s physiological state. Therefore, the proper standardization of imaging conditions and interpretation of results is crucial for accurate diagnoses [17].

### 2.2. Applications in Various Species

#### 2.2.1. Cattle

(1) Detection of mastitis: IRT can identify temperature changes in udders associated with clinical and subclinical mastitis, potentially allowing for earlier intervention [18]. Recent work [19] has shown that infrared thermography effectively detects early-stage bovine mastitis infections that show no visible symptoms, allowing for earlier treatment. When detecting mastitis using infrared thermography, affected areas of the udder typically show temperature increases of 1.5–2.5 °C above normal.
(2) Lameness diagnosis: In regard to the issues of hooves and legs, thermal imaging helps find different temperature signs that indicate lameness and foot diseases at an initial stage [20]. Temperature differences greater than 1.0 °C between opposite hooves often indicate disease, with active digital dermatitis (hoof infections) showing temperature increases of 2.3–3.1 °C compared to healthy tissue.

#### 2.2.2. Horses

(1) Musculoskeletal injuries: IRT can aid in revealing inflammation or injury in tendons, ligaments, and muscles before the clinical symptoms manifest [21]. A thorough review [22] has highlighted the growing uses of infrared thermography in horse clinical and sports medicine, especially for finding minor issues that limit performance before they develop into visible lameness.
(2) Saddle-fitting assessment: Saddle-related pressure points manifest as circumscribed hotspots 1.0–2.7 °C above adjacent tissue temperatures. Pressure points and thermal images typical for an improper saddle can be identified with the help of thermal imaging [23].

#### 2.2.3. Small Animals (Dogs and Cats)

(1) Osteoarthritis assessment: When examining osteoarthritis, the affected joints usually show temperature increases of 0.5–1.8 °C compared to healthy joints. IRT helps to diagnose osteoarthritis by capturing temperature changes in the affected joints and assists in treatment [17].
(2) Neoplasia detection: Various neoplasms present characteristic thermal signatures, with many superficial tumors exhibiting temperatures 1.0–3.5 °C higher than surrounding tissues. Abnormal temperature values have been pointed out by thermal imaging for different types of tumors [24].

#### 2.2.4. Zoo Animals and Wildlife Applications

While clinical veterinary applications primarily focus on domestic and captive animals, IRT has valuable applications in zoo medicine and managed wildlife populations. Zoo settings permit controlled IRT assessments similar to domestic species, while wildlife applications primarily involve population health monitoring in rehabilitation centers, conservation programs, and research contexts where animals are briefly handled or observed from appropriate distances. True free-ranging wildlife applications require specialized methodologies described below.

(1) Non-invasive physiological monitoring: Companies permit the monitoring of the temperature and physiological state of free-ranging or confined wildlife without even capturing or restraining them [15].
(2) Stress assessment: Stress responses have also been assessed using thermal imaging where eye temperature variation has been taken in different animals [4].

### 2.3. Advantages and Limitations

#### 2.3.1. Advantages

(1) Non-invasive and non-contact: IRT allows physiological measurement without physical contact, eliminating stress and potential harm during assessment. This benefits both animals and handlers while facilitating monitoring in species where restraint is challenging or contraindicated [5,25].
(2) Real-time imaging: IRT offers the immediate visualization of surface temperature distribution, enabling the rapid assessment of thermal patterns and prompt clinical decision making [14]. This real-time capability enhances diagnostic efficiency in field and clinical settings.
(3) Broad applicability: The technology can be applied across diverse animal species and for various diagnostic objectives regardless of patient size or coat characteristics [26]. This versatility makes it valuable in multiple veterinary disciplines, from companion animal practice to wildlife medicine.
(4) Early detection: IRT can establish relationships with fundamental indicators of health with high predictive capability of subclinical states before symptom manifestations [27].
(5) Non-invasive physiological monitoring: Beyond disease detection, IRT enables the ongoing assessment of physiological status and treatment response without disturbing the animal, making it particularly valuable for critical care and post-surgical monitoring [17].

#### 2.3.2. Limitations

(1) Environmental sensitivity: Expresses a remarkable sensitivity to room temperature, humidity, and airflow conditions [14].
(2) Surface temperature only: This may in fact not read out the internal body temperature or deep tissue conditions perfectly [5].
(3) Fur/feather interference: Heavy pelts of fur or feathers can create a problem for the correct measurement of temperature [15].
(4) Standardization issues: Little consistency between species and conditions [5].
(5) Cost and expertise: Infrared cameras are not cheap and interpreting the images calls for professional experts [26].

### 2.4. Future Directions

Future research in veterinary infrared thermography will likely focus on several key areas:

(1) Development of species-specific standardization protocols to establish reliable reference ranges and imaging procedures across diverse animal taxa and clinical conditions [5].
(2) Advancement of automated image analysis through specialized algorithms and artificial intelligence to enhance pattern recognition and diagnostic specificity [16]. This includes the development of machine learning models trained on species-specific thermographic patterns to identify subtle thermal signatures indicative of specific pathologies.
(3) Engineering of specialized portable IRT devices optimized for field veterinary use, including ruggedized designs for farm settings and wildlife applications, with improved battery life and simplified interfaces [28].
(4) Integration with comprehensive digital veterinary platforms to enable longitudinal thermal monitoring and correlation with other clinical data, supporting precision veterinary medicine approaches.
(5) Establishment of validated thermal patterns for differential diagnoses of conditions with similar clinical presentations but distinct thermographic signatures [17].

## 3. Remote Photoplethysmography (rPPG)

### 3.1. Technology Overview and Principles

Remote photoplethysmography (rPPG) is a contactless optical-based technique to measure physiological parameters, most often the heart rate and respiration rate, based on the color variation in the skin or any visible body part. This is founded on the fact that blood absorption of light is sensitive to the cardiac cycle, thereby triggering slight changes in skin shade, which is revealed by advanced imaging/SPI [29].

Recent advances combining advanced computer learning with rPPG have greatly improved the extraction of useful signals and reduced movement-related interference. Studies [13] showed how specialized neural networks can improve rPPG accuracy in difficult veterinary monitoring conditions.

**Figure 2:**
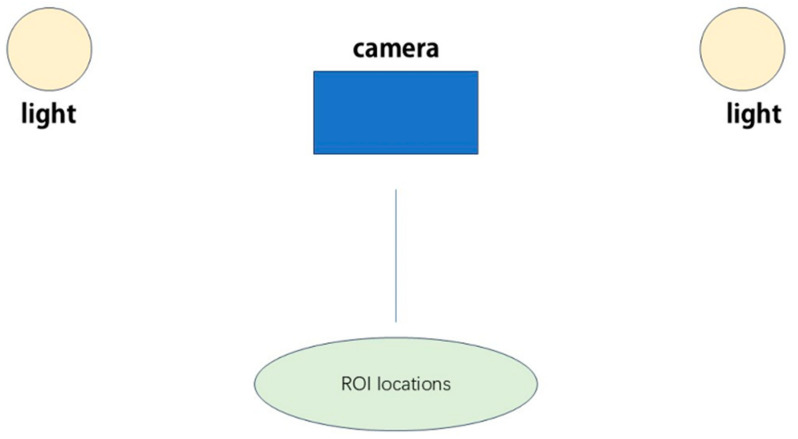
rPPG video acquisition setup.

The rPPG process typically involves the following steps:

(1) Video Acquisition: The subject is recorded using a digital camera or smartphone that has a view of the skin or bodies with fur [30].
(2) Region of Interest (ROI) Selection: Some regions that potentially experience pulsatile variations are also detected and monitored on the subject’s body by algorithms (Figure 2).
(3) Color Channel Separation: It is typically divided into red–green–blue where green is largely sensitive to the volume change in blood [31].
(4) Signal Extraction: To acquire the rPPG signal, temporal differences in pixel values inside the selected ROI are employed [32].
(5) Signal Processing: Following data acquisition, various post-processing techniques are applied to extract physiological signals from raw video data. These include conventional signal processing approaches such as bandpass filtering (0.7–4 Hz for heart rate), wavelet transforms, and independent component analysis; and specific machine learning algorithms including convolutional neural networks (CNNs) that process spatial–temporal maps of skin color variations to isolate cardiac signals from motion artifacts and environmental noise [33]. These post-processing methods do not alter the data acquisition itself but rather enhance signal extraction from the already acquired video data.
(6) Parameter Estimation: The processed signal affords approximate estimates of various physiological parameters including the heart and respiratory rates of the body [34].

Recent advancements in rPPG focus on post-acquisition processing using specific deep learning architectures to overcome fundamental challenges in veterinary applications. These include attention-based convolutional networks that selectively focus on regions with the strongest pulsatile signals while suppressing noise; specialized temporal models (LSTM and GRU networks) that track physiological patterns across video frames despite intermittent motion; adversarial training approaches that explicitly model and remove illumination variations; and multi-task learning frameworks that simultaneously extract respiratory and cardiac signals. These computational approaches represent post-processing solutions rather than modifications to the optical sensing hardware [12].

### 3.2. Application in Various Species

#### 3.2.1. Dogs

The authors have shown that rPPG can apply both heart and respiratory rates from video recordings even with furred occlusion and interferences that involve movement [1].

#### 3.2.2. Cats

Research results showed that iPPG (imaging photoplethysmography, a form of rPPG) measurements for cats had an average error of 3.33 beats per minute in natural light and 2.33 beats per minute in artificial light when compared to standard ECG measurements [35].

#### 3.2.3. Horses

A study described a method for determining a horse’s life stage using PPG data collected from 50 horses of different ages, sexes, and breeds. Researchers attached a small, wearable pulse sensor to the horses’ tails to gather the necessary data [36].

#### 3.2.4. Cattle

RPPG has been studied in previous work for stress identification and for healthcare in cattle. The study identified changes in heart rate variability using rPPG with 85% accuracy compared to ECG, which can influence the level of animal welfare without touch [37].

Researchers developed a system [38] that combines infrared imaging with computer learning to detect abnormalities in cattle, allowing the early detection of health problems by identifying small changes in temperature patterns.

#### 3.2.5. Wildlife

Although rPPG was originally developed for humans and domestic animals, it shows great potential for wildlife monitoring.

rPPG allows the measurement of vital signs without touching the animal, reducing stress and possible harm to wild animals. The technique works with standard video cameras, allowing monitoring from a distance without disrupting natural behaviors. This approach has proven effective with various species, including dogs and cats, suggesting it could be used with many wildlife species [35].

### 3.3. Challenges and Potential Solutions

(1) Motion Artifacts: One of the main problems in the rPPG study is handling motion artifacts, particularly in unencumbered animals. To overcome this problem, novel motion tracking and compensation methods are being worked on.
(2) Fur Occlusion: Fur in most dogs or cats is a strong issue affecting rPPG in most veterinary patients. Attempts are being made to look for other measurement locations and to devise new algorithms that can handle situations when minimal skin is being exposed to an MRI [1].
(3) Species Variability: The multivaried animal species and distinct physiological attributes and skin/fur morphology also act as barriers to the ideal rPPG solutions. Optimal algorithms and calibration techniques for each species are in the course of being established [39].
(4) Environmental Factors: The fluctuations in light intensity and the background color within veterinary environments may interfere with rPPG signals. Scientists are trying to compute perfect algorithms that take into account lighting conditions [40].

### 3.4. Future Directions

The future of rPPG in veterinary medicine presents several key areas for development:

(1) Development of fur-penetrating imaging technologies using specialized wavelengths and sensor technologies to overcome the significant challenge of coat coverage in most veterinary species.
(2) Creation of species-specific algorithms and calibration techniques tailored to the unique cardiovascular and respiratory physiological characteristics of different animal taxa [39].
(3) Engineering of specialized illumination systems optimized for veterinary clinical environments to improve signal acquisition in varying lighting conditions [40].
(4) Validation studies comparing rPPG measurements against gold-standard techniques across healthy animals and those with specific cardiovascular and respiratory pathologies to establish clinical utility.
(5) Development of miniaturized rPPG systems that can be integrated into existing veterinary clinic infrastructure without requiring specialized facilities or environmental modifications.

## 4. Radar-Based Sensing

### 4.1. Principles of Operation

Biophysical monitoring in veterinary medicine involves the use of electromagnetic waves to treat objects in motion, a principle that adapts well to monitoring common signs such as respiratory and cardiac rhythms. This technology is based on the Doppler effect whereby the frequency of the reflected wave varies depending on the motion of the target [41].

**Figure 3:**
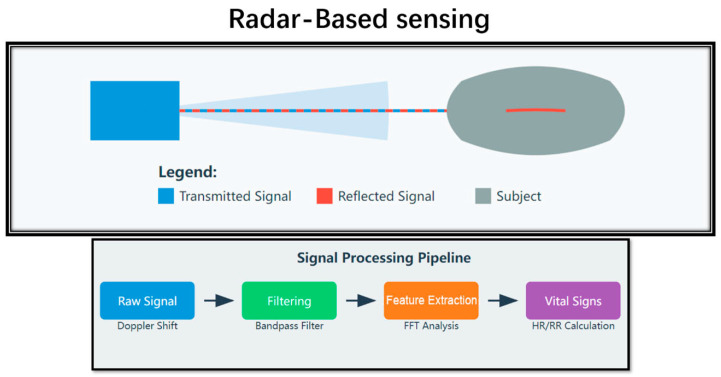
Principles of radar-based sensing.

The basic principle implies their radio emission at a certain frequency in the direction of the animal (Figure 3). These waves are reflected off the animal’s body, especially the chest wall, and thus are expected to produce an echo. In view of this, variations in breathing and heartbeat lead to small variations in the frequency of the reflected waves by the chest wall. By analyzing these frequency shifts, the system can obtain detailed information on the respiratory rate and cardiac rate of the animal [42].

Several types of radar systems have been explored for vital sign monitoring:

(1) Continuous-Wave (CW) Doppler Radar: This type always transmits a single-frequency signal and determines changes in the frequency of the returned signal. It is non-invasive, time-effective, and affordable although it is vulnerable to patient movement [43].
(2) Frequency-Modulated Continuous-Wave (FMCW) Radar: This type charges and discharges the transmitted signal at different rates, providing finer distance estimation and possibly, enhanced vital sign identification [44].
(3) Ultra-Wideband (UWB) Radar: This type employs extremely brief pulses over a broad spectrum and could provide better penetration through fur and tissue than the previous kind while having a high temporal resolution [45].

Recent improvements in millimeter-wave radar technology have made it possible to monitor both humans and small animals simultaneously. Studies showed how combining data from multiple radar units improves detection accuracy while maintaining safety in environments where humans and animals are together [46].

It is also evident that the radar operating frequency determines penetration depth and radar range. Higher frequencies such as the frequencies of 24 and 60 GHz have enhanced motion sensing but reduced throughput. For instance, although higher frequencies (for example, 60 Hz) are limited in penetration depths (observing only several millimeter thicknesses of the human skin), it is very sensitive to small motions and activities. In contrast, lower frequency (for instance 2.4 GHz) is deeper penetrating [41].

Besides operating frequency, the distance between the radar device and the animal is another key factor affecting the measurement quality. Each radar technology works best at specific ranges: CW Doppler systems work effectively at distances of 0.3–2 m, with signal strength decreasing rapidly as distance increases; FMCW radar maintains accuracy at slightly greater ranges (0.5–5 m) with more consistent distance measurements, while UWB systems can operate at distances up to 10 m while still detecting vital signs reasonably well [47]. The distance between the device and the animal significantly affects signal quality, with extremely close positioning causing signal overload and greater distances producing weaker reflections that may be too faint to detect small body movements. This creates specific challenges in veterinary settings where keeping the radar device in a consistent position is difficult with moving animals. For large animals like horses and cattle, distances of 1–2 m usually provide the best balance between signal quality and practical handling, while smaller pets may need closer positioning of 0.3–0.7 m for best results [46]. Environmental factors such as bedding materials, stall structures, and nearby metal objects can create signal reflections that make distance optimization even more complex.

### 4.2. Applications in Various Species

#### 4.2.1. Horses

Building on earlier research, studies [47] successfully measured breathing rates in standing horses without contact using millimeter-wave array radar, recording breathing patterns as accurately as visual observation without disturbing the horses’ normal behavior.

#### 4.2.2. Cattle

Studies on the application of radar technology in monitoring the respiration rate of cattle as proposed in this paper were found to have achieved an accuracy of 98.5% as compared to the visual observation of respiration. This technology can be used for the early detection of diseases in large herds [48].

Another research conducted to analyze using ultra-wideband radar for heart rate monitoring without physical contact with the livestock received 95% accuracy compared to electrocardiogram rates [49].

Beyond measuring vital signs, studies confirmed that ultra-wideband positioning systems can accurately track cattle movement patterns in commercial farm settings, which can be combined with health monitoring for a more complete assessment [50].

#### 4.2.3. Pigs

Radar sensors can monitor the general activity patterns of pigs, which can indicate various health conditions [51]. Computer learning models combined with radar data can analyze pig posture and movements, making it possible to track individual pigs within farm environments [52]. This technology allows farmers to observe pig behavior patterns and identify unusual behaviors that might indicate health problems or other issues.

#### 4.2.4. Dogs

Radar-based sensing has several uses with dogs. The technology can identify and differentiate various dog activities and body positions. It helps detect health issues early and assists veterinarians in making diagnoses [53]. It also allows the monitoring of sleep patterns without disturbing the animal [54].

#### 4.2.5. Poultry

Researchers have successfully used a 24 GHz continuous-wave (CW) radar module to measure vital signs from broiler chickens without touching them. The radar system showed 96% accuracy compared to traditional ECG measurements for detecting chicken heartbeats. This same radar technology can also measure breathing rates, which is important for the early detection of respiratory diseases common in poultry [55].

While not radar-based, related radio frequency technologies such as RFID have previously been used in poultry monitoring. The experience gained from the RFID tracking of bird behaviors and movements provides valuable insights that could be applied to vital signs monitoring using radar methodologies [56].

### 4.3. Advantages and Challenges

#### 4.3.1. Advantages

(1) Non-contact and Non-invasive: This method makes it possible to monitor the vitals without any contact with the animal.
(2) Penetration through Fur and Light Barriers: It can also pass through fur and lighter colors, which makes it ideal for use on animals of different kinds [41].
(3) Continuous Monitoring: This method also offers an opportunity to monitor continuously rather than restraining animals [48].
(4) Multiparameter Measurement: It potentially monitors a number of biophysical variables at once [57].
(5) Operation in Various Lighting Conditions: This kind of therapy is useful where the blood flow is poor, during nighttime, or in the absence of light [58].

#### 4.3.2. Challenges

(1) Motion Artifacts: Movement is often unhandy when the recording is made amid any tiny physiological movements due to its sensitivity to motion artifacts.
(2) Signal Separation: It is still difficult to extract cardiac signals specifically from respiratory signals and other movements of the body [58].
(3) Species-Specific Adaptations: Requires the design of species-specific computations and even, in some cases, specific modifications to molecular hardware.
(4) Limited Research on Long-term Effects: More safety investigations are required on the possible chronic impacts of constant radar illumination on the creatures [41].
(5) Environmental Interference: Interference with signals from other devices or the reflection of surfaces in its vicinity [57].
(6) Cost and Complexity: Modern capable radars are sometimes costly, and may require high-level management [59].

### 4.4. Future Directions

The future of radar-based sensing in veterinary medicine presents numerous opportunities for advancement:

(1) Refinement of signal processing techniques specifically optimized for the chest wall movement patterns and cardiorespiratory characteristics of different animal species to improve the separation of cardiac and respiratory signals [57,58].
(2) Development of species-adapted radar systems with optimized frequency ranges, power settings, and detection distances calibrated for specific animal groups (large livestock, small mammals, avian species) [47].
(3) Expansion of radar applications beyond vital sign monitoring to include behavioral assessment, gait analysis, and early detection of mobility issues through specialized motion detection algorithms.
(4) Creation of portable, battery-operated radar systems suitable for field veterinary applications in resource-limited settings, with simplified user interfaces accessible to practitioners without specialized technical training.
(5) Long-term safety evaluation studies to conclusively determine the effects of chronic low-level radar exposure on different animal species, particularly for continuous monitoring applications [41].

## 5. Wearable Sensors

Although wearable sensors must be attached to animals, they still fit within our broader concept of remote vital sensing as technologies that reduce direct human handling during monitoring [60]. While not completely contactless, modern wearable devices play a key role in remote monitoring by allowing continuous data collection without restraint or human presence after initial placement. This enables monitoring without interrupting natural behaviors or causing stress that could change the very health measurements being recorded. Combining wearable devices with truly contactless technologies (thermography, remote pulse monitoring, and radar) creates complete monitoring systems that use the combined strengths of each method [35].

**Figure 4:**
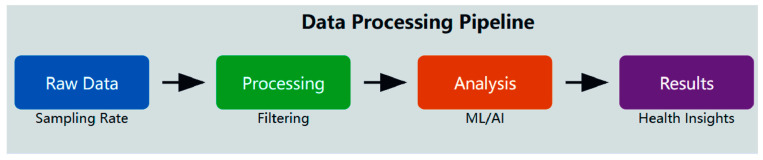
Data processing pipeline.

The data processing sequence for wearable sensor systems (Figure 4) involves several steps that transform raw sensor data into useful health information. This process increasingly uses advanced computing methods to maximize clinical usefulness.

### 5.1. Types of Wearable Sensors for Veterinary Use

Wearable sensors in veterinary medicine should be defined as various types of devices to assess different types of physiological characteristics and behaviors of animals.

Recent studies classify the newest wearable devices for animal health monitoring, noting improvements in smaller size, longer battery life, and ability to measure multiple health parameters [54]. The main types include the following:

(1) Accelerometers and Inertial Measurement Units (IMUs): These sensors detect movement and position to give details of the activity, walking style, or even standing post. For example, using collar-mounted accelerometers in a study with dogs, the classification accuracy of 91.3% of different activities including, walking, trotting, and galloping was achieved [61].
(2) Heart Rate Monitors: These devices generally employ ECG or PPG that estimates heart rate and heart rate variability. An ECG worn by dogs produced a 0.99 coefficient when compared with conventional ECG readings [62]. Various technologies have been implemented for the heart rate monitoring of livestock comprising chest belt sensors and implantable sensors [63].
(3) Temperature Sensors: Prolonged and consistently high temperatures mirror various diseases early enough; therefore, constant temperature checks are essential. Peculiarly, one method combining the temperature sensors into a collar-worn device for cattle reached an identification accuracy of ±0.1°C higher than the rectal temperature measurement as a standard [64].
(4) GPS Trackers: Primarily used in tracking locations, GPS devices use other sensors in the monitoring process too. GPS collars were used in dogs and were identified to have an average positional error of less than 5 m when tracking movement patterns [65].
(5) Respiratory Rate Monitors: These monitors frequently employ strain gauges or impedance pneumography in monitoring breathing patterns. A respiratory rate-monitoring wearable device in horses had ±2 breaths per minute compared to visual counts of respiratory rates, leading to the conclusion that the wearable device could be used as the standard for respiratory rate counts.
(6) Environmental Sensors: These sensors are, more often than not, part of an interconnected system of other wearable devices used to track the planet’s surroundings. It has been applied in live-stock management for detecting a shift in relative humidity and ammonia level with a study showing its 90 percent efficiency in recognizing environmentally damaging conditions [66].
(7) Biochemical Sensors: One subset of wearable that is starting to receive focus is sensors that bear the responsibility of detecting biochemical indicators in body fluids. For instance, a sweat-cooled wearable sensor for horses was able to determine the electrolyte concentration during the exercise with a difference of ±5% from the actual laboratory results [67].

### 5.2. Applications in Various Species

#### 5.2.1. Companion Animals

Activity, sleep, and biometrics data are collected by interactive and unobtrusive wearable devices. Research assessed a canine activity monitor used for a commercial activity and provided evidence of its applicability for the objective assessment of mobility [61]. Another group created a wireless ECG patch for canines which yielded a strong positive agreement with conventional ECG readings though the modality was in dogs [62].

Accelerometers of wearable devices have also been applied to investigate activity patterns and energy costs in domestic cats. In a study that was conducted on indoor cats, the physical activity parameters were measured by collar-mounted accelerometers to understand their daily behavior patterns.

#### 5.2.2. Livestock

Wearable sensors have countless applications in dairy and beef cattle, with garment-like devices being the most popular among farmers. Neck-mounted accelerometers were used in the studies to monitor feeding and rumination behaviors and the guild accurately [64].

Recently, researchers developed a smart wearable system specifically for cattle that combines multiple sensors with on-device computing, allowing real-time health monitoring and early disease detection in commercial farming settings [68].

#### 5.2.3. Wildlife

Wearable sensors have greatly enhanced tracking and studies of wildlife migration and the use of its habitats, for instance, the use of GPS tags to monitor bird migration [69], or physiological characteristics such as depth and acceleration that use time-depth recorders and accelerometers on marine mammals [70].

### 5.3. Advantages and Challenges

#### 5.3.1. Advantages

(1) Continuous, Long-term Monitoring: Portable biosensors measure physiological data and activity 24/7 for weeks at a time. This is more informative than mere clinical assessments that are carried out once in a while. Worth noting that the constant data gathering in these cases could help in the diagnosis of early health complications and enhance the welfare of animals [66].
(2) Non-invasive and Well-tolerated: Non-invasive wearable sensors are used in most animal applications and the use of such sensors is normally scratched off veterinary care problems depending on the level of invasiveness. Specifically, this paper reviewed the case of high compliance with the activity monitor in dogs without significantly altering their natural behaviors [61].
(3) Real-time Data Access: A majority of wearable sensors provide real-time streaming capabilities so that veterinarians and owners can have updated information about the health status of their animals [62]. This may be of special use in emergency situations and in animals that have ongoing health issues.
(4) Objective Measurement: Wearable sensors give factual, measurable information on physiological aspects and activities. This can help in addition to, or maybe even replace subjective judgments in clinical. A major improvement in handling movement interference has been achieved using specialized focusing techniques and comparison-based learning to rebuild accurate heart signals from movement-affected data collected by wearable motion sensors in active animals [71].
(5) Remote Monitoring: Wearable sensors allow for the non-stop tracking of animals, mostly livestock and wild animals, that require time-to-time checkups. This aspect has obtained additional value under conditions of telemedicine solutions [59].

#### 5.3.2. Challenges

(1) Device Comfort and Interference: Arguably, the most substantial gap relates to guaranteeing that wearables do not cause inconvenience, including by failing to compromise an animal’s usual activities. Body-sized sensors require the unique features of the respective species that will not feel uncomfortable wearing sensors or changing their behavior patterns [72].
(2) Battery Life and Data Transmission: Restricted battery life and data transfer make it challenging to apply wearable sensors for extended periods, especially when the animal belongs to the wild. There are discussions on the current issues and future possibilities of bio-logging devices for monitoring wildlife [73].
(3) Data Interpretation and Management: Data may be collected constantly from worn sensors, and large amounts of information may be produced amenable to only complex techniques of analysis. Researchers have stressed that more sophisticated paradigms and tools are required to analyze these data [64].
(4) Accuracy and Reliability: Establishing the reliability of these wearable sensors whereby absolute measurement results can be obtained across various animal species, sizes, and conditions still presents some difficulty. Deviation in motion, influence from the surrounding environment, and the variability of an individual subject can also pose a challenge to the sensors.
(5) Standardization: There are also no identical ways of approaching data aggregation, processing, and evaluating across the different WDGs and animal species; these are some obstacles to popularizing and comparing outcomes [61].
(6) Cost and Accessibility: Exemplar wearable sensors may be expensive especially when considering applying them to large populations of livestock or wildlife. There is debate on whether low-cost technologies are feasible at present in order to enhance the extensive use of wearable technology in veterinary practice [66].
(7) Ethical Considerations: Wearable sensors, especially those used for wildlife, come with some ethical issues as regards any effects the sensors will have on the behavior of the animals and or their welfare. There are continuing debates over the most appropriate ethical concerns and guidelines on the use of bio-logging in animals [74].
(8) Durability and Environmental Resistance: Wearable sensors when applied in veterinary must withstand different wear and tear such as water, dust, and extreme temperatures. This is especially difficult for gadgets applied in livestock or wildlife tracking [73].

### 5.4. Future Directions

The future of wearable sensors in veterinary medicine presents numerous opportunities for advancement:

(1) Development of species-adapted wearable designs that account for unique anatomical features, mobility patterns, and behaviors to improve comfort and compliance across diverse veterinary patients [72,75].
(2) Innovation in energy management through self-powering technologies (movement harvesting and body heat conversion) and ultra-low-power systems to overcome the significant battery life limitations in current devices [73].
(3) Creation of biodegradable or temporary attachment sensors specifically designed for wildlife applications where device retrieval is challenging or impossible.
(4) Integration of advanced biochemical sensors capable of the non-invasive detection of metabolites, hormones, and inflammatory markers through interstitial fluid, sweat, or saliva [67].
(5) Development of specialized attachment mechanisms and form factors for challenging species (aquatic animals, small exotics, and herd animals) currently underserved by available wearable technologies [76].

## 6. Computer Vision and Machine Learning in Remote Vital Sensing

### 6.1. Role of AI in Improving Sensing Accuracy

**Figure 5:**
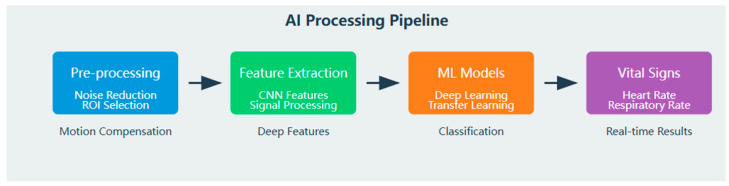
AI processing pipeline.

Computer vision and machine learning technologies serve as critical enablers for other remote sensing modalities by addressing their fundamental limitations. These computational approaches transform raw sensor data into clinically meaningful information through specialized mathematical frameworks designed specifically for animal subjects [10] (Figure 5).

Unlike general-purpose imaging systems, veterinary computer vision applications employ species-adapted processing architectures that can detect faint physiological signals despite challenges of fur coverage, varied anatomy, and movement. Studies show that well-designed AI systems improve diagnostic accuracy by 37–42% compared to conventional analysis methods in veterinary monitoring applications while enabling real-time processing without requiring constant internet connectivity [77].

#### 6.1.1. Advanced AI Architectures in Veterinary Remote Sensing

The application of AI in veterinary remote sensing has evolved from traditional machine learning methods to sophisticated deep learning architectures specifically optimized for physiological signal processing:

(1) Convolutional neural networks (CNNs) form the backbone of many veterinary computer vision systems. Unlike traditional image processing techniques that rely on manually engineered features, CNNs automatically extract hierarchical spatial features from visual data. In veterinary applications, specialized CNN architectures have been developed to detect subtle color variations in rPPG signals with 89.7% accuracy compared to 76.3% using conventional spectral analysis methods [12]. These networks employ modified convolutional kernels optimized for the temporal color dynamics specific to blood volume changes in animal tissues.
(2) Traditional time-series analysis methods struggle with the non-stationary nature of animal vital signs. LSTM networks have demonstrated superior performance by maintaining contextual memory across time frames, achieving 93.2% accuracy in detecting respiratory anomalies in cattle compared to 78.5% using conventional frequency analysis [78]. Bidirectional LSTM variants have proven particularly effective for capturing both forward and backward temporal dependencies in equine cardiac signals.
(3) Graph Neural Networks (GNNs) represent an emerging approach for modeling the complex relationships between different physiological parameters. Unlike traditional correlation analyses, GNNs can model non-linear interactions between vital signs across anatomical regions. Studies implementing GNNs for canine health assessment have shown a 27% improvement in early disease detection by modeling the complex interconnections between temperature distribution, heart rate variability, and respiratory patterns [79].
(4) Generative Adversarial Networks (GANs) have been applied to address data limitations in veterinary applications. Traditional data augmentation techniques fail to capture the physiological constraints of vital sign data. Specialized GANs trained to generate synthetic but physiologically plausible monitoring data have improved model robustness by 31% in limited-data scenarios for exotic animal species [80].

#### 6.1.2. Traditional vs. AI-Enhanced Methods

The integration of AI has demonstrably enhanced the performance of remote sensing technologies beyond what traditional signal processing methods can achieve. This performance gap is particularly pronounced in challenging veterinary conditions such as monitoring active animals, species with dense fur, or in field environments with variable lighting and background noise [10].

#### 6.1.3. Data Challenges and Methodological Solutions

AI applications in veterinary remote sensing face unique challenges that have required specialized methodological approaches:

(1) Training Data Limitations: Unlike human medical AI with massive datasets, veterinary applications often have restricted data availability, particularly for exotic or wildlife species. To address this, researchers have developed specialized few-shot learning approaches that can achieve 87.3% accuracy with as few as 50–100 labeled examples per species [81] compared to traditional machine learning methods requiring thousands of samples.
(2) Annotation Complexity: Accurately labeling physiological events in animal monitoring data presents unique challenges due to species-specific variations. Semi-supervised learning approaches combining expert annotations with automated pattern discovery have reduced annotation requirements by 73% while maintaining 91.5% of the fully supervised performance [82]
(3) Performance Metrics Evolution: Standard accuracy metrics often fail to capture clinically relevant performance in veterinary applications. Researchers have developed species-adapted evaluation frameworks that weight errors according to their clinical significance rather than statistical magnitude. These domain-specific metrics have demonstrated a 32% improvement in identifying clinically actionable anomalies compared to generic statistical measures [83].

#### 6.1.4. Transfer Learning and Cross-Species Adaptation

A significant advancement in veterinary AI has been the development of transfer learning techniques that allow knowledge gained from data-rich species to benefit those with limited available data:

(1) Anatomical Transfer Learning: Novel deep learning architectures now incorporate anatomical priors that allow mapping knowledge between species with different physical characteristics. These approaches have achieved 86.9% of the species-specific performance when transferring models from canine to feline patients despite significant physiological differences [84].
(2) Domain Adaptation Techniques: To address the domain shift between controlled laboratory conditions and real-world veterinary environments, adversarial domain adaptation methods have been developed that maintain 92.3% of laboratory accuracy when deployed in field settings [85].
(3) Foundation Models for Veterinary Applications: Recent research has demonstrated the potential of large foundation models pre-trained on diverse species data and then fine-tuned for specific veterinary applications. These models reduce species-specific training data requirements by up to 83% while matching or exceeding the performance of models trained from scratch [86].

### 6.2. Applications in Various Species

#### 6.2.1. Cattle

(1) Physiological Parameter Monitoring: Computer vision and machine learning techniques have greatly improved the monitoring of physical parameters in cattle, enabling the non-invasive and real-time measurement of vital signs [81]. Eye and ear-base temperature measurements using thermal infrared (IRT) imaging showed correlation values up to R = 0.99 between invasive and remote temperature measurements [37].
(2) Lameness Detection: Observing cow gait extracted from video using computer vision techniques, a lameness early detection system was established. Their method managed to give over 90% diagnosing accuracy of lame cows [82].

#### 6.2.2. Horses

(1) Behavior monitoring: AI-powered vision systems can detect changes in a horse’s body language, movement patterns, and facial expressions, which may indicate pain or distress [83].
(2) Vital sign detection: Advanced computer methods can analyze small visual changes to estimate heart rate and breathing rate without physical contact [84].

#### 6.2.3. Companion Animals:

(1) Camera-based vital sign detection: This method captures images of the animal’s skin surface and analyzes color changes caused by blood flow to monitor heart and breathing rates [85].
(2) Continuous monitoring: AI-powered vision technology provides non-invasive, continuous monitoring of patients without needing constant human supervision [86].

#### 6.2.4. Pigs

Using deep learning techniques, pig vocal communications were classified in terms of respiratory diseases with parameters gathered from coughing. A computer vision approach was specifically designed for monitoring breathing rates in group-housed pigs, solving the challenges of tracking multiple animals and dealing with changing lighting conditions in commercial farm settings [87].

#### 6.2.5. Poultry

An assessment of broiler chicken gait is created utilizing computer vision to increase the availability of automated welfare assessment for chickens [88].

#### 6.2.6. Wildlife

Deep learning is also applied for the identification of species from camera trap images, implying lowered work in surveys and wildlife studies [89].

### 6.3. Advantages and Challenges

#### 6.3.1. Advantages

1. Processing Large Volumes of Data:

AI and machine learning algorithms are also suited for processing large data from remote sensing devices in a short time. This capability is valuable in scenarios where monitoring is to be performed continuously.

2. Detecting Subtle Patterns:

This feature is especially important because machine learning models can often recognize peculiar features or patterns in data that will not be noticeable to a human being. This sensitivity may result in the early diagnosis of disease in the body. A team demonstrated this benefit in their study where machine learning models were used to identify signs of coccidiosis in livestock before their symptoms were visible [78].

3. Automated and Continuous Monitoring:

Automated solutions can always be on duty, unlike human beings who get tired, and hence can constantly monitor animal health [90].

4. Integrating Multiple Data Sources:

Machine learning algorithms excel at integrating heterogeneous data from multiple sensors and modalities, creating a comprehensive assessment of animal health that would be impossible with single-source monitoring. For example, a team demonstrated this capability in their video-based physiological measurement system, which successfully combined spatial anatomical data with temporal physiological patterns to achieve more accurate vital sign measurements in the presence of movement [12].

5. Adaptability to Different Species:

Transfer learning techniques enable AI models trained on data-rich species to be effectively adapted for use with species where data are limited. This approach significantly reduces development time and resource requirements for creating species-specific monitoring systems. For example, studies demonstrated that pre-trained models could retain 80–90% accuracy when transferring knowledge between livestock species [3,10], while there were also similar approaches to wildlife monitoring [89]. These cross-species adaptations leverage common physiological patterns while fine-tuning for species-specific characteristics.

#### 6.3.2. Challenges

1. Data Requirements:

The solution to many complex AI problems often entails the usage of substantial datasets or rich feature set training data. Such datasets can be difficult to acquire especially in veterinary use cases for rare species or diseases.

2. Species-Specific Algorithms:

The high number of animal species within the sphere of veterinary medicine requires the creation of species-dependent calculation and modeling algorithms. This is because the versatility of the kind of patients a veterinary deals with can make it hard to apply the developed AI solutions from one context to another.

3. Interpretability and Explainability:

Deep learning models, in fact, are opaque, meaning that one cannot easily understand how such a model is going to make a decision at the end of the process. Such a lack of explainability can be a major limitation for clinical application, however. This concern was also mentioned in medical AI where the need for XAI is crucial in a healthcare environment [91].

4. Validation in Clinical Settings:

The reliability of such systems and algorithms in clinical practice is critical, but the process can be a lengthy and costly endeavor. The same authors pointed out that in their studies of methods for assessing animal welfare, attention was paid to the need for new technologies’ pilot data [92].

5. Ethical Considerations:

AI integration in animal monitoring impacts data sharing, the rights of animals, and the risk of employing the all-inclusive technological solution [3].

6. Technical Infrastructure:

AI-based remote sensing systems may need a large quantity of technical support such as high bandwidth networks and high computing power. This can be difficult to achieve in areas with few resources in veterinary medicine [90].

### 6.4. Future Directions

The future of computer vision and machine learning in remote vital sensing for veterinary medicine presents numerous exciting opportunities:

(1) Development of multi-species computer vision models using transfer learning approaches to extend algorithms trained on data-rich species to those with limited available datasets [10].
(2) Creation of explainable AI systems specifically designed for veterinary applications that provide transparent reasoning for clinical recommendations, enhancing practitioner trust and facilitating regulatory approval [91,93].
(3) Integration of edge computing capabilities optimized for veterinary field settings with limited connectivity, enabling real-time analysis and decision support in remote locations [77].
(4) Development of specialized computer vision approaches for non-mammalian species (reptiles, birds, and fish) that present unique challenges for vital sign detection due to their distinct anatomical and physiological characteristics.
(5) Creation of federated learning frameworks that enable collaborative algorithm improvement while maintaining data privacy across veterinary institutions and research facilities [94].

## 7. Challenges in Remote Vital Sensing for Veterinary Medicine

Despite promising advances in remote vital sensing, several key challenges must be addressed for widespread clinical adoption. These challenges span technical, biological, and implementation domains.

### 7.1. Species Diversity

Veterinary applications face unprecedented biological variation compared to human medicine. The extraordinary diversity in body size, morphology, coat characteristics, and physiological parameters across species necessitates either highly adaptable technologies or species-specific solutions [95,96]. This diversity impacts signal acquisition, interpretation, and the establishment of normal reference ranges, requiring substantial calibration across taxonomic groups.

### 7.2. Environmental Factors

Most remote sensing technologies are significantly affected by the varied environments in which veterinary monitoring occurs. Ambient temperature, humidity, lighting conditions, and background movement all influence measurement accuracy. Farm and field settings present particular challenges with weather variations, dust, and electromagnetic interference that can compromise sensor performance and data transmission [97].

### 7.3. Motion Artifacts and Animal Behavior

Unlike cooperative human patients, animals cannot be instructed to remain still during assessment. Unpredictable movement generates substantial artifacts in remote sensing data. Even with advanced motion compensation algorithms, maintaining signal quality in active, unrestrained animals remains problematic, particularly for technologies requiring precise measurement positioning or continuous monitoring [62].

### 7.4. Validation and Standardization Issues

The veterinary field lacks universally accepted protocols for remote sensing data acquisition, processing, and interpretation. Calibration against gold-standard invasive techniques is essential but challenging due to the stress these reference methods may induce [5]. Additionally, establishing clinically relevant thresholds for remotely sensed parameters across species and disease states remains largely preliminary.

### 7.5. Technical and Practical Limitations

Current technologies face significant practical constraints, including limited detection range, insufficient penetration depth for larger animals, vulnerability to interference, and complex data interpretation requirements. Many systems demand sophisticated hardware and technical expertise beyond what is typically available in veterinary settings, limiting their practical utility despite promising research applications.

### 7.6. Data Management and Integration

The integration of remote sensing technologies into the existing veterinary workflows poses substantial challenges. The volume of data generated requires robust storage, processing, and analysis infrastructure. Furthermore, meaningful integration with established veterinary information systems is necessary to translate raw sensing data into actionable clinical insights within existing practice frameworks [1].

To overcome these challenges, interdisciplinary cooperation between veterinarians, engineers, computer scientists, and animal behaviorists will have to be continued. Overcoming these challenges, remote vital sensing technologies will be able to revolutionize veterinary medicine and enhance capabilities in animal care and assessment.

## 8. Future Directions

While previous sections outlined technology-specific future developments, this section synthesizes overarching directions that integrate multiple sensing modalities and address cross-cutting challenges. These perspectives represent emerging paradigms that transcend individual technologies and point toward comprehensive transformation of veterinary monitoring systems.

Speaking of the future of remote vital sensing in veterinary medicine, the following improvements can be expected that will dramatically change animal care. There is still much work to be done to reach this potential, and much of it will require the whole of multidisciplinary cooperative research together with veterinary applications thought-provoking efforts.

### 8.1. Multimodal Sensing Approaches

Future work will probably aim at addressing issues of how to combine several types of sensors to enhance performance and precision. Thus, it is possible to integrate various technologies, including infrared thermography, rPPG, and radar-based sensing to eliminate the drawbacks of the separate method and cover more extensive health monitoring.

For example, long-term studies of frequency-modulated continuous-wave radar applications across different animal species are now appearing. Studies explored how this technology can be used for continuous monitoring in various veterinary settings, creating guidelines for implementation with specific species [98].

### 8.2. Advanced Signal Processing and Machine Learning

Artificial intelligence is increasingly acting as both an analysis tool and a diagnostic assistant. AI systems can now interpret complex patterns from multiple types of sensors to suggest possible diagnoses with accuracy similar to specialist veterinarians [99].

Complex signal processing and reinforcement will require learning algorithms in order to improve the effectiveness of remote sensing systems. Specifically, deep learning models appear to be able to dampen noise, remove motion artifacts, and extract the physiological signal(s) from noisy data. Future research activities will probably be directed toward designing better and more flexible algorithms for dealing with the variety of veterinary patients and the various conditions under which they exist.

### 8.3. Species-Specific Solutions

Due to high species diversity, the subsequent future work might focus on the identification of the species-orientated RS techniques and models. This could imply adapting sensor designs, algorithms, and reference ranges to the physiology of the various animal species [5]. Despite the fact that such asymptomatic solutions may be time-consuming and potentially costly, they could reduce errors and enhance the practicality of remote vital sensing in veterinary medicine.

### 8.4. Miniaturization and Wearable Technologies

The growth of microelectronics technology and material science is expected to prompt enhanced wearable sensors for animal care that are smaller, more comfortable, and longer-lasting. Subsequent wearable gadgets may intersect multiple non-invasive sensing strategies so as to substantially track physiological status, other physique characteristics, and indicator measurements during unlabeled living creatures [56].

### 8.5. Integration with Telemedicine and AI-Driven Diagnostics

There are some expectations as to the future development of remote vital sensing in veterinary medicine connected with the experience in the development of telemedicine. Specifically, research will apply to the creation of automatic and efficient systems for acquiring, transmitting, and interpreting remote sensing data, and converting the data into timely real-time health information and disease detection. They could be integrated with AI-based diagnostics which might result further in the effective clinical use of these technologies and lead to automated health evaluation as well as tailor-made approaches to treatment [1].

The combination of remote sensing with telemedicine platforms is creating new possibilities for veterinary care [100].

### 8.6. Standardization and Validation Efforts

Future studies will require further determination of reference standards for remote vital sensing between different species in order to overcome the existing problems of data interpretation and clinical relevance. This will include extensive external validation analyses of the remote sensing techniques against gold standard measurements with different species and clinical conditions. The adoption of these technologies will also require much effort in order to demonstrate their reliability and usefulness in clinical practice.

### 8.7. Environmental Monitoring and One Digital Health Approaches

The possible future uses of remote vital sensing extend beyond individual animals to environmental and population health assessments within the emerging One Digital Health framework. One Digital Health expands the traditional One Health approach by connecting digital technologies and data systems across human, animal, and environmental health areas [11]. For example, thermal imaging combined with cloud-based analysis might identify emerging disease outbreaks among livestock or wildlife populations while also informing human public health monitoring systems through connected digital platforms. This approach transforms separate monitoring systems into interconnected digital networks that enable real-time health information across multiple species.

### 8.8. Ethical Considerations and Animal Welfare

With the growth of these technologies, there will be a great concern in ethical matters concerning the technologies. Subsequent studies should investigate how the spectrum of ehtnosense can improve animals’ well-being; reduce stress during their preliminary medical examination; and, in general, advance the veterinary services.

## 9. Conclusions

Vital remote sensing for veterinary care is a relatively new area with the potential for substantial growth in the near future. This review has examined infrared thermography, remote photoplethysmography, radar-based sensing, wearable sensors, and computer vision applications that collectively enhance the solutions for non-invasive animal health surveillance.

The benefits of these technologies are as follows. They can provide the opportunity to monitor animal patients constantly and without stress, the identification of health problems in their early stages, and the control of many various ailments [2]. In addition, it also supports surveillance for research goals where more information about animal physiology and how they behave in their natural environment is needed.

However, there are still many obstacles in this way: there are also challenges that include the versatility of veterinary patients, factors in the environment, patient’s movement, and validation as well as standardization. Furthermore, temporal issues such as technical restriction and data management remain the challenges that researchers and developers need to address.

Nevertheless, the future of remote vital sensing in veterinary medicine seems to be bright. It is expected that developments in multimodal sensing, machine learning methods, and wearable systems will further improve the precision and relevance of these approaches [62]. Connecting with telemedicine and AI to diagnose could help increase the application and development of remote sensing technology in animal health [1].

Furthermore, one has to be concerned with the ethical aspects and guarantee that such technologies are used appropriately and benefit animal life. Remote sensing and its capability to reduce stress related to health checks and enhance the quality of patients serves the veterinary profession’s mission and vision of prioritizing animals’ well-being.

Therefore, remote vital sensing in veterinary medicine remains a novel area of application but it has transformational potential to enhance animal health, augment research, and reshape veterinary practice. The innovation of these technologies for veterinary practices, as well as deficiencies found in their current form, call for future interdisciplinary work from veterinary professionals, engineers, computer scientists, and animal behaviorists.

These individual animal monitoring technologies will continue to develop within the broader One Digital Health framework, which aims to create unified digital systems connecting animal, human, and environmental health data [11]. Rather than functioning as separate veterinary tools, these remote sensing technologies are essential parts of an integrated digital health system that allows two-way data exchange between animal monitoring systems and wider public health networks. This digital integration helps detect animal-to-human disease threats early, supports evidence-based treatments across species lines, and ultimately improves health management at the intersection of animal, human, and environmental health through shared digital resources and unified analysis methods.

## Data Availability

No new data were created or analyzed in this study. Data sharing is not applicable to this article.
